# Identification of region of difference and H37Rv-related deletion in *Mycobacterium tuberculosis complex* by structural variant detection and genome assembly

**DOI:** 10.3389/fmicb.2022.984582

**Published:** 2022-09-08

**Authors:** Zhuochong Liu, Zhonghua Jiang, Wei Wu, Xinyi Xu, Yudong Ma, Xiaomei Guo, Senlin Zhang, Qun Sun

**Affiliations:** ^1^Key Laboratory of Bio-Resources and Eco-Environment of the Ministry of Education, College of Life Sciences, Sichuan University, Chengdu, China; ^2^College of Biomass Science and Engineering, Sichuan University, Chengdu, China

**Keywords:** *Mycobacterium tuberculosis* complex, large sequence polymorphism, region of difference, H37Rv-related deletion, structural variant

## Abstract

*Mycobacterium tuberculosis* complex (MTBC), the main cause of TB in humans and animals, is an extreme example of genetic homogeneity, whereas it is still nevertheless separated into various lineages by numerous typing methods, which differ in phenotype, virulence, geographic distribution, and host preference. The large sequence polymorphism (LSP), incorporating region of difference (RD) and H37Rv-related deletion (RvD), is considered to be a powerful means of constructing phylogenetic relationships within MTBC. Although there have been many studies on LSP already, focusing on the distribution of RDs in MTBC and their impact on MTB phenotypes, a crumb of new lineages or sub-lineages have been excluded and RvDs have received less attention. We, therefore, sampled a dataset of 1,495 strains, containing 113 lineages from the laboratory collection, to screen for RDs and RvDs by structural variant detection and genome assembly, and examined the distribution of RvDs in MTBC, including RvD2, RvD5, and *cobF* region. Consistent with genealogical delineation by single nucleotide polymorphism (SNP), we identified 125 RDs and 5 RvDs at the species, lineage, or sub-lineage levels. The specificities of RDs and RvDs were further investigated in the remaining 10,218 strains, suggesting that most of them were highly specific to distinct phylogenetic groups, could be used as stable genetic markers in genotyping. More importantly, we identified 34 new lineage or evolutionary branch specific RDs and 2 RvDs, also demonstrated the distribution of known RDs and RvDs in MTBC. This study provides novel details about deletion events that have occurred in distinct phylogenetic groups and may help to understand the genealogical differentiation.

## Introduction

*Mycobacterium tuberculosis* (MTB) and other members of the *Mycobacterium tuberculosis* complex (MTBC) are responsible for the development of tuberculosis (TB) in humans or animals. In addition to MTB, MTBC also comprises *M. africanum* (Vasconcellos et al., 2010), *M. canetti* (Soolingen et al., 1997), *M. orygis* (Pittius et al., 2012), *M. caprae* (Niemann et al., 2002), *M. bovis* (Garnier et al., 2003), *M. mungi* (Al, 2010), *M. suricattae* (Parsons et al., 2013), *M. microti* (Brodin et al., 2002), and *M. pinnipedii* (Cousins et al., 2003). With the exception of *M. canetti*, the remaining members of MTBC have selfsame 16S rRNA sequences and nearly identical genome sequences, and there is no horizontal gene transfer between MTBC strains, therefore MTBC is regarded as a conspicuous example of remarkable genetic homogeneity, and the extreme similarity proves that they share a common ancestor, but still differ in host preference, phenotype, and virulence (2003; Hirsh et al., 2004; Sanou et al., 2015). Among them, MTB and *M. africanum* are the most common causes of TB in humans, and prior studies have classified them into 9 primary lineages (lineage 1∼9), some of which are strongly geographically restricted (lineage 1, 3, and lineage 5∼9), while others (lineage 2, 4) are found globally distributed (Coscolla and Gagneux, 2014; Devis et al., 2020; Ngabonziza et al., 2020).

Several methods have been developed for molecular typing of MTBC strains, including IS6110 restriction fragment length polymorphism (IS6110 RFLP), mycobacterial interspersed repetitive units-variable number tandem repeats (MIRU-VNTR), and spacer region oligonucleotide typing (Spoligotyping). These methods have showed the high resolution and perform well in clinical testing, traceability, and re-infection detection. However, exorbitant diversity or, in some cases, excessive homogeneity has led to the application of these methods in phylogenetic analysis not entirely suitable for constructing reasonable phylogenetic relationships for MTBC. In addition to the molecular typing methods described above, Napier et al. (2020) provided a typing scheme based on single nucleotide polymorphism (SNP) consisting of 90 specific robust SNPs to cover 90 MTBC (sub-) lineages or species; although the SNP-defined lineages do not offer the same resolution as using the whole genome, they provide a valuable insight into the epidemiology of circulating strains.

Early comparative genomics studies used whole-genome microarrays or bacterial artificial chromosomes to identify areas with considerable diversity in the genomes of distinct MTBC lineages, known as large sequence polymorphism (LSP), which are frequently deleted in different strains (Brosch et al., 1998; Gordon et al., 1999). The associated deletion events are often unidirectional and irrecoverable, which will be inherited by all progeny strains. The LSPs are thus regarded as a valuable tool for establishing plausible phylogenetic relationships within MTBC, in addition to being utilized for molecular typing. LSPs incorporates region of RD and RvD, where RD refers to deletions relative to H37Rv and RvD refers to deletions in H37Rv relative to the rest of the strains.

Whole-genome sequencing (WGS) has achieved advancements in the study of MTB resistance, transmission kinetics, and phylogenetic analysis of MTBC as sequencing technology improves (Meehan et al., 2019). RD-Analyzer was the pioneer in the analysis of LSPs using WGS data to predict the species or lineage of MTBC strains based on 31 previously defined RDs (Kiatichai et al., 2016). Previous studies of RDs, as observed by Shitikov (Bespiatykh et al., 2021), have focused on discovering RDs within certain MTBC lineages, frequently without addressing the remaining members of the MTBC, or on studying the relationship of various members within the MTBC, although often only considering crucial genomic regions. Therefore, only a small number of RDs are available, and the distribution of RDs in MTBC lacks comprehensive understanding. Shitikov compiled a complete map of RDs in MTBC by collecting known RDs, examining the specificities of these RDs in a dataset encompassing several MTBC lineages, and identifying novel RDs. In addition, they developed RDscan, a pipeline for detecting RDs using WGS data.

In contrast with RDs, RvDs have received less attention using WGS data, possibly because of the discovery that RvDs frequently require genome assembly. Early studies identified that 5 RvDs (RvD1 ∼ RvD5) were present in *M. bovis* but absent in H37Rv, and it was suggested that the absence of RvD2 ∼ RvD5 could be due to recombination between adjacent isotropic IS6110 (Brosch et al., 1999; Gordon et al., 1999), increasing the difficulty of finding RvDs in assembled draft genomes because of the need to overcome the gap introduced by IS6110. Aside from the deletion of TbD1 in modern lineages and the retention of the *cobF* region in Lineage 8 and *M. canetti*, few studies have found that RvDs are linked to genealogical differentiation in MTBC (Brosch et al., 2002; Ngabonziza et al., 2020).

We sampled a dataset of 1,495 strains from the laboratory collection of MTBC strains with WGS data from 113 lineages, and RDs were screened using a pipeline consisting of multiple tools to detect deletions, while RvDs were screened by genome assembly. We identified 125 RDs and 5 RvDs, incluing 34 newly named RDs and 2 RvDs, specific to distinct phylogenetic groups. In addition, we discovered a complicated deletion in the RvD4496 region in the genome of lineage 5, where the RvD4496 was partially deleted and a 3.5 kb long fragment was absent in the downstream region. Most of the identified RDs and RvDs were highly specific to distinct phylogenetic groups and could be used as stable genetic markers in genotyping according to the results of specificity test in remaining strains. Further, we demonstrated the distribution of known RDs and RvDs in MTBC to show the details about deletion events, and this may help to understand the genealogical differentiation within MTBC.

## Materials and methods

### Dataset

WGS data for 11,713 MTBC strains were collected from the NCBI-SRA archive.^1^ The SRA files were downloaded locally using sratoolkit (version: 2.11.3)^2^ before being decompressed into pair-end FASTQ files. All WGS data obtained were quality controlled using Fastp (version: 0.23.1) (Chen et al., 2018). After mapping to reference genome, depth and sequencing coverage of WGS data were counted by bamdst,^3^ and those with depth below 50 × and coverage below 90% were excluded. Then after SNP typing, a random sample of 1,495 strains from 113 lineages constituted the dataset used for screening RDs and RvDs. The work flow of the analysis process used for the sampled dataset and the remaining strains could be referred to Supplementary material 9.

### SNP calling and typing

A pipeline built in-house in the laboratory was applied to perform variant calling for all strains (reference genome H37Rv, NCBI accession number: GCF_000195955.2). The pipeline was as follows: quality-controlled WGS data were mapped to the reference genome using bwa-mem2 (version: 2.2.1) (Vasimuddin et al., 2019), before being sorted by samtools (version: 1.14) (Danecek et al., 2021) and PCR duplicates were marked using sambamba (version: 0.8.0) (Tarasov et al., 2015), respectively, followed by bamdst (version: 1.0.9) (see text footnote 3) to calculate the sequencing depth and coverage, and removed strains with below 90% coverage. SNP typing was performed using fast-lineage-caller (version: 0.3.2)^4^ and a Python script based on the method of Clark (Napier et al., 2020). Only strains with consistent typing results in both methods were retained for subsequent analysis. Strains of lineage 1.1.1, lineage 2.2.1, lineage 5, and lineage 6 were classified into more specific sub-lineages according to the methods of Shitikov (Shitikov et al., 2017), Palittapongarnpim (Palittapongarnpim et al., 2018), and Coscolla (Devis et al., 2020). Labels in the NCBI-SRA archive of the strains belonging to animal-adapted lineages were reserved.

### Phylogenetic analysis

All SNP loci of all strains were sequentially aligned and each strain was filled at these loci with deletion substitutions of “–,” while loci with deletion rates higher than 20% were removed to produce the final aligned fasta files. SNPs in PE/PPE family genes, known drug resistance genes, and non-SNP variation were removed. Possible tandem repeat regions in the H37Rv were identified by TRF (version: 4.09)^5^ and SNPs located in these regions were removed. The phylogenetic tree was constructed by RAxML (version: 8.0.0) (Stamatakis, 2014) using a maximum likelihood method with 500 bootstraps, and finally visualized and modified using iTol.^6^

### Structural variant detection

Delly (version: 0.8.7) (Rausch et al., 2012), Manta (version: 1.6.0) (Chen et al., 2016) and SvABA (version: 1.1.3) (Wala et al., 2018) were used to detect deletions in strains with default parameters. Results of a single strain were merged by SURVIVOR (version: 1.0.7) (Jeffares et al., 2017), to combine deletions detected by different cnallers with breakpoints located within 200 bp of each other, and deletions greater than 200 bp by more than two callers were retained. Genome assembly was performed using Shovill (version: 1.1.0)^7^ with default parameters, followed by SVIM-asm (version: 1.0.2) (Heller and Vingron, 2020) to detect structural variants and recorded deletions and insertions greater than 200 bp. Among the results, those associated with DR regions (direct repeat regions, cluster and regularly spaced CRISPR sequences for spolygotyping) were removed.

### Re-genotyping and structural variation filtering

All structural variants detected in individual strains were collected and merged by SURVIVOR to form a structural variant library and used for subsequent re-genotyping of all strains to reduce false negatives in individual strains. Possible differences in breakpoints between individual strains were temporarily ignored, and the exact breakpoints of these structural variants will then be confirmed manually with IGV (version: 2.11.2) (Robinson et al., 2011). Re-genotyping was performed for all strains using svtyper (version: 0.1.1) (Chiang et al., 2015). For deletions, the following steps were performed to filter the re-genotyping results to reduce false positives and to ensure that deletions occurred mainly in single copy regions. Specifically, bamdst was employed to count sequencing uncoverage for calculating proportion of uncoverage (ratio of the total length of the sequencing uncoverage within deletion to the total length of the deletion), and deletions were considered as true positives when the proportion of uncoverage was higher than 0.75. Deletions with overlapping range may interfere with the filtering, so the proportion of uncoverage within 200 bp flanking the deletions was examined to determine if extended deletions existed. Deletions were considered to be extended when the proportion of uncoverage within 200 bp flanking the deletion was higher than 25%. For insertions, insertion fragments caused by large fragment duplications were removed by sequence characterization to ensure that the insertion fragment was novel. Specifically, the specific sequences of insertions with relevant insertion sites (located within 200 bp from each other) were collected, and multiple sequences alignment were performed to confirm whether they were likely to be the same insertion and to obtain concordant sequences, followed by searching for similar sequence fragments in the reference genome using MMseqs2 (version: 13-14511).^8^ The longest segment in “Query coverage” was found from the BLAST results with “Percent of Identity” higher than 75%, and the product of “Percent of identity” and “Query coverage” of this segment was taken as the total similar sequence proportion. When the proportion was inferior to 0.75, the insertion fragment was considered as a novel insertion. Then a reference sequence containing all novel insertions was constructed separately to determine their presence by checking the sequencing coverage. More precisely, same as checking whether deletions were true positive, we mapped the sequencing data to the reference sequence containing the novel insertions, and then calculated the sequencing coverage for each novel insertion by bamdst. When the proportion sequencing coverage was above 0.75, we assumed the presence of the novel insertion in the strain.

### Screening for region of differences and H37Rv-related deletions

The number of strains with certain deletions or insertions in each lineage was counted and the RDs and RvDs were screened based on the following criteria: when the number of strains in the lineage was less than 10, all strains should have the deletion, and when the number of strains was greater than 10, a maximum of 10% and no more than 5 strains were allowed to be free of the deletion (as these could be sequencing errors or false negatives). Later, we will examine the distribution of screened RDs and RvDs in MTBC to confirm whether they converge in specific lineages or phylogenetic clades.

### Breakpoint confirmation

A custom Python script was used to determine if both ends of the RDs were located in or near tandem repeat regions. For each RD and RvD, breakpoints were confirmed as follows: mapping the draft genome assembled by Shovill to H37Rv using minimap2 (version: 2.24) (Li, 2018), generating different SAM files by lineage, sorting and converting to BAM files using samtools. Then, IGV was used to visualize BAM files and the exact breakpoints of the deletion or insertion were set as those with the highest frequency. And for breakpoints located in repeat regions, the exact breakpoints were set as those with the largest deletion range.

### Structural variation annotation and covariance analysis

For RDs, genes included in deletions were identified by a custom Python script to confirm whether the deleted genes were essential according to DeJesus (DeJesus et al., 2017). For RvDs, covariance analysis was performed including H37Rv, lineage 8 (NCBI accession number: GCF_012923765.1), lineage 5 (NCBI accession number: GCF_905183075.1), and *M. canetti* (NCBI accession number: GCF_000253375.1). Sequences flanking the insertion site of RvDs were obtained by bedtools (version: 2.28.0) and then mapped to the rest of other reference genomes using minimap2 to generate SAM files. The SAM files were viewed through Pycharm, the best mappings were recorded as covariance regions, and the sequences of the covariance regions (insertion fragments and regions near the insertion sites) were obtained using bedtools. The genes contained within the covariance regions were identified by comparing the annotation files (GFF files) of each reference genome, before being aligned between the reference genomes by MMseqs2 to confirm the covariance of genes. Concordant sequences of RvDs reported by previous studies but not detected by Shovill and SVIM-asm were also acquired by covariance analysis, including RvD2, RvD5, and *cobF* regions (Brosch et al., 1999; Ngabonziza et al., 2020), and the reference genome of *M. bovis* (NCBI accession number: GCF_005156105.1) was additionally used here.

## Results

### Dataset and phylogenetic analysis

Variant calling and SNP typing were performed on the laboratory collection of 11,713 MTBC strains, and a dataset consisting of 1,495 strains from 113 lineages was randomly sampled (Supplementary material 2). There were less than ten strains in this dataset for 22 lineages, including several lineages with sub-lineages such as lineage 3.1, lineage 4.1, lineage 4.3, lineage 4.4, lineage 4.6, and lineage 4.6.1, but only one strain for *M. mungi*. The rest of the other lineages contained 11∼31 strains. Since lineage 1.3 and its sub-lineage were not found in the laboratory collection, they were not included in the sampling dataset.

**FIGURE 1:**
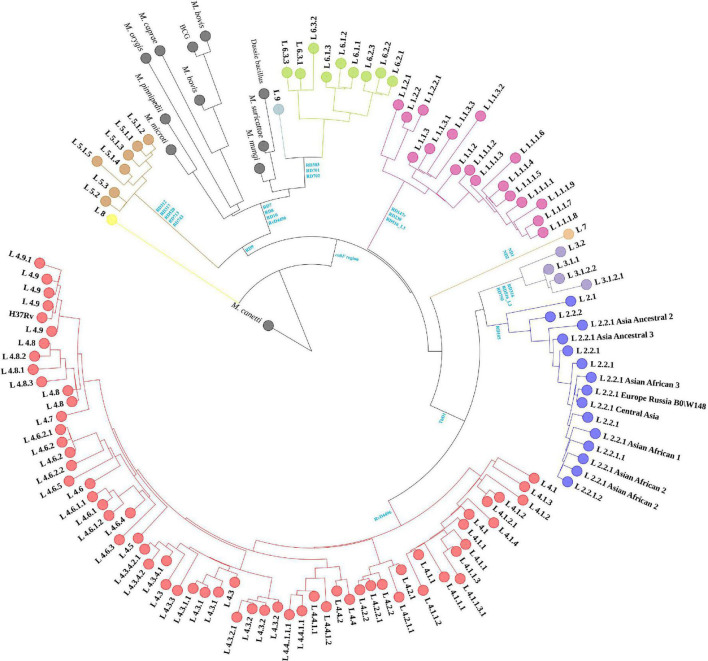
Maximum-likelihood phylogenetic tree of MTBC strains in sampled dataset. Clades were shrunk by lineage or sub-lineage, and the size of external nodes did not represent the number of strains.

The 1,495 MTBC strains’ phylogenetic relationships were inferred from 146,872 SNPs, with *M. canetti* as the root (Figure 1). The branch lengths of *M. canetti* were manually truncated to show the phylogenetic relationships and branch lengths of the remaining members in the MTBC. The recently discovered lineage 8 was thought to be separated from the remaining MTBC members before they diverged because of the presence of the *cobF* region (Ngabonziza et al., 2020). Phylogenetic analysis verified the phylogenetic status of lineage 8. Apart from lineage 8, the remaining MTBC members were divided into two main evolutionary branches, one for human-adapted lineages (lineage 1∼4 and lineage 7), and the other for the traditionally known *M. africanum* (lineage 5, 6, and lineage 9) and the animal-adapted lineages. A new lineage of *M. africanum*, lineage 9, was identified by Devis et al. (2020) as a sister lineage to lineage 6. Previous studies have split the animal-adapted lineages into four distinct evolutionary branches, A1 (*M. suricattae*, *M. mungi* as well as “chimpanzee” and “Dassie” bacillus, but “chimpanzee” bacillus was not included in this study), A2 (*M. microti* and *M. pinnipedii*), A3 (*M. orygis*) and A4 (*M. caprae* and *M. bovis*) (Brites et al., 2018). In the sampled dataset, the strains labeled as these species were easily distinguishable. Overall, phylogenetic analysis revealed that phylogenetic connections were mostly consistent with earlier research (Brites et al., 2018; Napier et al., 2020).

### Structural variant detection, re-genotyping and filtering

**FIGURE 2:**
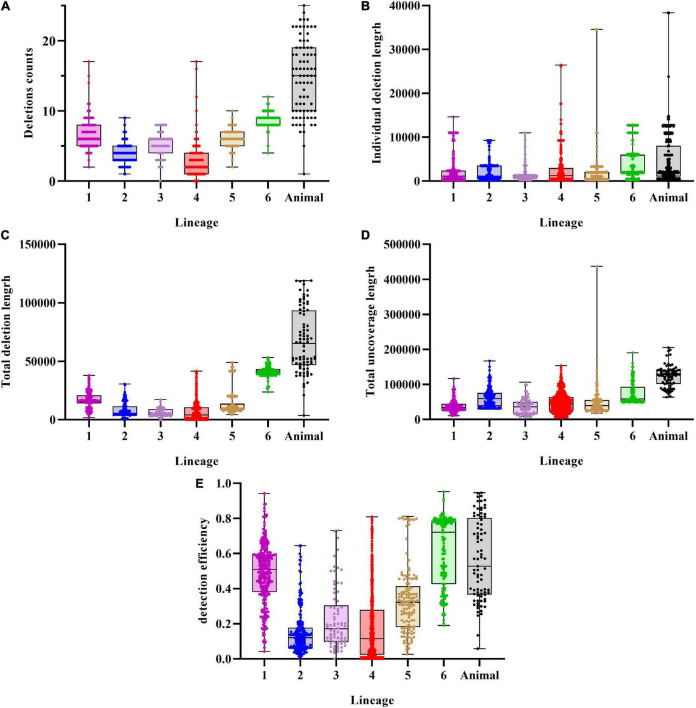
Characteristics of deletions in MTBC strains. **(A)** Deletions per genome distribution among MTBC strains. **(B)** Individual deletion length distribution among lineages. **(C)** Total deletions length per genome distribution among MTBC strains. **(D)** Total uncoverage length per genome distribution among MTBC strains. **(E)** Detection efficiency of deletions distribution among MTBC strains.

Prior to re-genotyping using svtyper, the effectiveness of the structural variation detection pipeline, including the number of deletions detected in a single strain, the length of an individual deletion, the total length of detected deletions, the total length of sequencing uncoverage, and the detection efficiency of deletions (the detection efficiency of deletions is the ratio of the total length of detected deletions to the total length of sequencing uncoverage), was calculated (Figure 2). We also applied a filtering threshold of 0.75 for the proportion of sequencing uncoverage inside the deletions to guarantee that the discovered deletions were true positive.

An average of 5.26 (*SD* = 3.68) deletions per strain was observed, with the animal-adapted lineage having the greatest average number of deletions per strain (14.74, *SD* = 5.31) and lineage 4 having the lowest (2.67, *SD* = 1.84). Individual deletions were on average 3,073 bp long, with lineage 6 having the greatest (4,686 bp), followed by animal-adapted lineages (4,573 bp), and lineage 3 having the lowest (1,112 bp). The structural variation identification method discovered an average total length of 16,179 bp per strain, with lineage 3 having the least (5,960 bp) and the animal-adapted lineage having the highest (67,412 bp). For total sequencing uncoverage, bamdst detected an average of 54,293 bp per strain, with lineage 1 having the least (37,518 bp) and animal-adapted lineages having the highest (123,145 bp). The maximum of 118,985 bp of deletions in total length was detected in one *M. caprae* strain and the maximum of 437,036 bp of sequencing uncoverage in total length was detected in one lineage 5.1.4 strain. The deletion detection efficiencies ranged from 0 to 95.15%, and the average was only 31.43%.

### Lineage specific region of differences and H37Rv-related deletions

**FIGURE 3:**
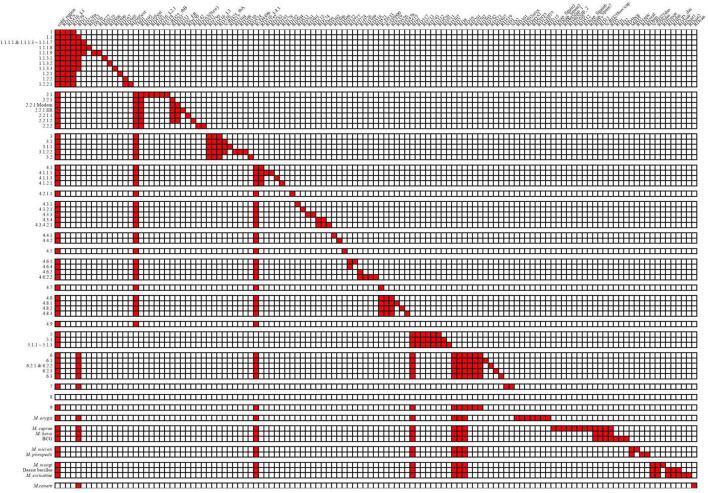
Deletion patterns of RDs and RvDs within MTBC.

A total of 125 RDs and 5 RvDs were screened for specific lineages or evolutionary branches (Figure 3), with 91 RDs and 3 RvDs already reported by previous studies (Supplementary material 3). The 34 newly identified RDs belonging to MTBC members other than *M. canetti* and lineage 8 were named, while the 2 newly defined RvDs were named RvD533 and RvD4496 based on their length. In all, 54 RDs belonged to the evolutionary branch of *M. africanum* and animal-adapted lineages, while the remaining 70 RDs correspond to *M. tuberculosis*. A total of 17 newly designated RDs MTBC were determined to be specific to 17 lineages for which no RDs had previously been found, such as lineage 4.2.1.1, lineage 4.6.3, lineage 1.2.2.1, and lineage 6.2.3.

According to its discoverer, the recently discovered genealogy, lineage 9, contains a deleted region spanning from Rv1762c to Rv1765, since Rv1763 and Rv1764 are presumptive IS6110-4 in H37Rv (Devis et al., 2020). In the region upstream of Rv1762c, Rv1755 ∼ Rv1757 are putative IS6110-3. Lineage 2, a sub-lineage of lineage 4, lineage 5, and *M. orygis*, had a high frequency of deletions in the region between and downstream of the two IS6110s, whereas *M. orygis* had a distinct range of deletions (Supplementary material 4—Figure 1). Previous studies have noted deletions in this region including RD152 (Tsolaki et al., 2004) and RD14 (Gordon et al., 1999), however, no deletions in this region have been identified by the structural variation detection pipeline in our study. We discovered that lineage 9 and its sister evolutionary branch, lineage 6, shared the identical pattern of RDs, with no RDs unique to each. However, the independent absence of RD11 (prophage phiRv2) in several lineages might be used to distinguish between the two, with all lineage 6 strains deleting RD11, while this deletion was detected in one lineage 9 strain (four in total) and additional Lineage 9 strains were needed to be confirmed whether the deletion of RD11 was common. We were only able to detect the specificities of these RDs belonging to lineage 8 since there were only two strains of lineage 8 in the dataset. We detected 5 of the 8 non-IS6110 deletions identified by the discoverer of lineage 8, with the exception of RD3 as well as the RD14 region, all of which differed from the previously identified range. In addition, we identified 5 additional RDs, but their authenticity and specificity need to be further determined.

Of the 5 RvDs, RvD1, TbD1, and the *cobF* region have been confirmed by previous studies (Gordon et al., 1999; Brosch et al., 2002; Ngabonziza et al., 2020). RvD1 was confirmed to be lacking in lineage 4.8 and lineage 4.9 in our study by checking its distribution in MTBC. Consistent with previous studies, TbD1 was absent in strains of the modern lineage (lineage 2∼4) and the *cobF* region was absent in all strains except *M. canetti* and lineage 8. RvD533 was deleted in lineage 4.7, lineage 4.8, and lineage 4.9. RvD4496 was absent in lineage 4, lineage 6, lineage 9, and animal-adapted lineages. Two other RvDs, RvD2 and RvD5, missing due to IS6110 recombination (Brosch et al., 1999), were examined for their distribution in MTBC (Supplementary material 3) (the congruent sequences of RvD2 and RvD5 in different genomes please see Supplementary material 5). RvD2 was shown to be deleted independently in multiple lineages, with some lineages having all strains lacking RvD2 and others having only some strains deleted RvD2, demonstrating that RvD2 is not a suitable lineage-specific RvD. In contrast to RvD2, RvD5 was only missing in partial strains within lineage 4.9, which is the closest lineage to H37Rv. In a previous study, in another reference genome candidate, H37Ra, also belonging to lineage 4.9, RvD5 was not missing (Brosch et al., 1999).

### Different types of deletions

Depending on the nature of the deletion and the presence or absence of repeat sequence or mobile elements at either end, deletions could be divided into three categories: deletions of mobile elements, for example, prophages and IS6110; deletions with mobile elements or repeat sequence at either end and those without repeat sequence or mobile elements at either end. The first two represent unstable regions of the genome.

The 2 prophages in H37Rv, phiRv1, and phiRv2, referred to as RD3 and RD11, respectively, in the RD system, were thought to be independently absent in different lineages (Fan et al., 2016). The structural variation detection pipeline we adopted allowed us to detect the deletion of RD3 and RD11 and to obtain precise breakpoints. In total, RD3 was deleted in 905 strains from 97 lineages. While the deletion of RD11 was detected in all lineage 1.1, lineage 6, lineage 7, animal-adapted A3, and A4 evolutionary branches. Outside of these lineages, the deletion was only detected in 41 samples from 13 lineages. Therefore, RD11 is still considered a stable RD for the above-mentioned lineages. IS6110 is a multi-copy mobile element in the genome and deletions of IS6110 at a single locus does not result in sequencing uncoverage. So that, in our study, deletions or insertions of IS6110 itself were ignored.

In addition to RD3 and RD11, by comparing to the result of TRF and the annotation of reference genome, we analyzed the presence of repeat sequence and mobile elements at either end of the remaining deletions to distinguish between the latter two deletion types. Only 8 breakpoints in 7 RDs were found in repeat sequence or mobile elements, while repeat sequence or mobile elements were present within 200 bp at either end of another 23 RDs, including deletions in the RD5 region and RD1 region. Deletions in the RD5 region were of more concern in this category, which was thought to result in reduced virulence in humans, and inconsistent ranges have been observed in different animal-adapted lineages (Ates et al., 2018; Supplementary material 4—Figure 2). The remaining 94 RDs did not have any repeat sequence or mobile elements at either end.

With the exception of RvD2 and RvD5, the five RvDs mentioned above had no tandem repeat sequences or mobile elements near the insertion sites. Although we could not find an exact insertion site of the *cobF* region, and we checked its probable insertion region (105.3 kb).

### Deletions with overlapping range

**FIGURE 4:**
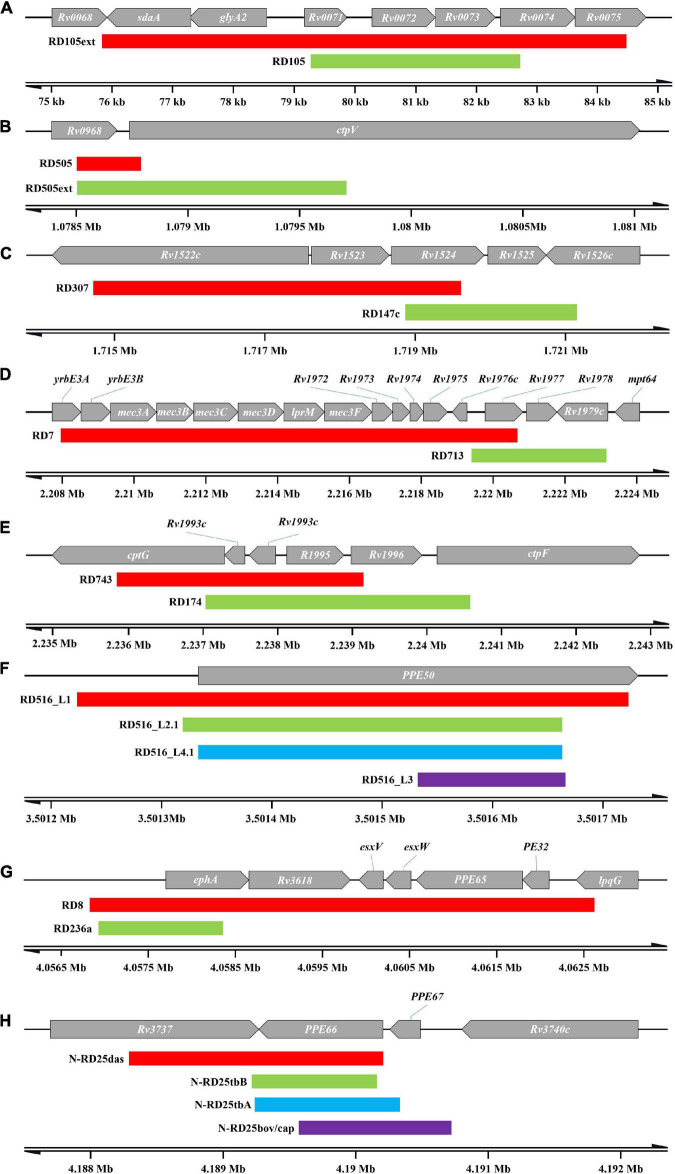
Overlapping RDs. Only the RDs mentioned in the text are shown, and do not include RD317 and RD306, and the deletion in RD5 region and RD1 region. **(A)** RD105 and RD105ext. **(B)** RD505 and RD505ext. **(C)** RD307 and RD147c. **(D)** RD7 and RD713. **(E)** RD 743 and RD174. **(F)** RDs in RD516 region. **(G)** RD8 and RD236a. **(H)** RDs in N-RD25 region.

19 groups of 57 RDs with overlapping ranges were identified (Figure 4), some of which may be associated with lineage differentiation. We could assume that different lineages, in which overlapping ranges of deletions occurred, if they could be divided into monophyletic groups in the phylogenetic tree, suggest that deletions of overlapping range may have occurred in a common ancestor, followed by different deletion extensions during subsequent divergence. For example, RD105 and RD105ext, classical RDs of lineages 2.2 and 2.1, respectively, with RD105ext having an extended deletion range to both sides compared to RD105. It is possible that deletion of RD105 was taken place in the common ancestor of lineage 2, and the extension occurred to form RD105ext during the differentiation into lineage 2.1 (Figure 4A). The same may also take place in RD505 and RD505ext (Figure 4B), RD7 and RD713 (Figure 4D), the deletions in RD5 region (Supplementary material 4—Figure 1) and the deletions in RD1 region (Supplementary material 6—i). RD505 and RD505ext are specific to lineage 1.2.2 and its sub-lineage 1.2.2.1, respectively, and their positions at the 3′-terminus are identical, but the deletion extended to form RD505ext in the further differentiated lineage 1.2.2.1. RD7 and RD713 are specific to two evolutionary branches, the evolutionary branch consisting of lineage 6, lineage 9, and animal-adapted lineages, and the evolutionary branch containing lineage 5, respectively. However, only a limited range of overlap existed between the two. In BCG, deletion extended at one end of RD5, while RD5 its self-corresponds to *M. bovis* and *M. caprae*. RD5oryx corresponds to *M. orygis*, however, the position of the 5′-terminus appears to change significantly amongst strains. Among the A1 evolutionary branch of “Dassie” bacillus, *M. mungi* and *M. suricattae*, deletions of the RD5 region appear to be more complicated. RD5das corresponds to *M. mungi* and was found in 2 of the 3 “Dassie” bacillus strains, while RD5sur, with a longer deletion range, corresponds to *M. suricattae* but was also found in 1 “Dassie” bacillus strains. The last group is the RD1 region with deletions RD1mon and RD1das. RD1mon corresponds to *M. mungi*, while RD1das corresponds to “Dassie” bacillus and *M. suricattae*. Although both BCG and *M. microti* have deletions in RD1 region, it was clear that they belong to different evolutionary branches from “Dassie” bacillus, *M. mungi* and *M. suricattae*, and did not constitute a monophyletic group.

The remaining RDs with overlapping ranges (Supplementary material 6) were identified in significant different evolutionary branches. For example, RD317 (lineage 5) and RD306 (lineage 4.4.1), RD307 (lineage 5.1.1 ∼ 5.1.3) and RD147c (lineage 1) (Figure 4C), RD743 (lineage 5) and RD174 (lineage 4.3.4) (Figure 4E), RD8 (lineage 6, lineage 9 and animal-adapted lineages) and RD236a (sub-lineages of lineage 1.1.1 except lineage 1.1.1.2) (Figure 4G). The N-RD25 (Figure 4H) area and the PPE50 gene (Figure 4F) are two other sites where deletions are linked to various lineages. N-RD25tbA corresponds to lineage 3; N-RD25tbB corresponds to lineage 2.1; N-RD25bovis/caprae corresponds to *M. bovis* and *M. caprae*, and N-RD25das corresponds to animal-adapted A1 evolutionary branch. There are four deletions in the PPE50 gene. RD516-L1 corresponds to lineage 1; RD516-L2.1 corresponds to lineage 2.1; RD516-L3 corresponds to lineage 3, and RD516-L4.1 corresponds to lineage 4.1.

**FIGURE 5:**
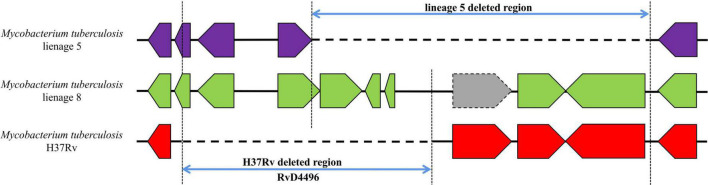
Gene covariation of RvD4496 region in lineage 5, lineage 8 and H37Rv. Gray block represents pseudogenes.

For RvDs, when performing covariance analysis (Supplementary material 7), we identified a complicated deletion in RvD4496 region in the genome of lineage 5 (Figure 5 and Supplementary material 3—**Table 1**). RvD4496 is partially deleted (the second half of the 5′-terminus), with deletion extending downstream to a 3.5 kb fragment in H37Rv.

### The importance of missing genes

The importance of the genes affected by RDs was confirmed in comparison to the study of DeJesus, although only for *in vitro* culture (Supplementary material 8). Only two genes (*Rv2017* and *Rv3902c*) were identified as essential among the 324 genes partly or totally eliminated owing to RDs (except RD528). *Rv2017* is presumed to be a transcriptional regulator. The function of *Rv3902c* is unknown, but studies suggest that it may be associated with virulence by co-expression with *EsxF*, *EsxE*, and *Rv3903c* (Danilchanka et al., 2014).

### Examining the specificity of region of differences and H37Rv-related deletions

We examined the specificities of these deletions in the remaining laboratory collection of 10,218 strains by assessing sequencing coverage (Supplementary material 3). 41 RDs were absent in strains outside of the specific lineages (excluding RD11, and deletions occurring in strains only classified into the upper lineage are also not counted). Since we were not concerned with the breakpoints in the genome of these strains, it could not be determined whether the exact range of these deletions was consistent with the RDs in range. Deletion of the RD5 region was detected in up to 168 strains of non-animal-adapted lineages; the next most common deletion was RD701, which was detected in 81 strains outside of lineage 6 and lineage 9; the remaining RDs were detected in no more than 35 strains outside of the non-specific lineage. Some of the RDs linked with the animal-adapted A3 and A4 evolutionary branches, such as those specific to *M. orygis* and *M. caprae*, respectively, were missing in some strains labeled as *M. bovis*, which might be due to labeling mistakes. *M. caprae* and *M. orygis* are closely related to *M. bovis*, hence in the early studies, they were often referred to as *M. bovis* until they were clearly identified. For the 5 RvDs, deletions were detected among non-specific lineages, except for *cobF* region.

**FIGURE 6:**
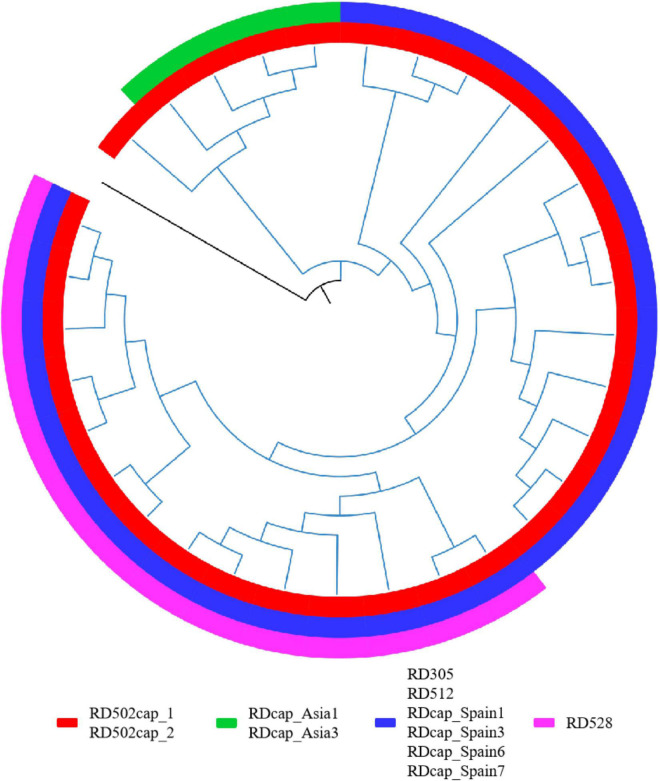
RDs in *M. caprae*. The blue branch represents the *M. caprae* strains and the black is *M. canetti* as the root. RD502cap_1 and RD502cap_2 are missing in all strains and the remaining RDs are only deleted in partial strains. RD528 is the largest deletion in length (38,328 bp) detected in this study and is deleted in 10 strains within the sampled dataset. Moreover, deletion of RD528 is detected in 4 additional strains out of all *M. caprae* strains.

In addition, some of the RDs might not be at the same node as the specific SNPs used for SNP typing, as evidenced by the detection of deletions in strains classified as upper lineage to the specific lineage, or the presence of true-negative non-deleted strains in the specific lineage. Further phylogenetic analysis of these strains is required to determine whether the RDs correspond to the specific SNPs. The RDs associated with *M. caprae* were the most prominent, and we performed a phylogenetic analysis of the laboratory collection of 32 *M. caprae* strains (Figure 6) in the same way as we did for other strains, confirming the occurrence of these RDs follows a pattern.

## Discussion

Despite the use of several detection modalities, the results demonstrated a poor detection efficiency, which might be attributed to the intrinsically short read length of next-generation sequencing. Multiple algorithms for structural variant detection using next-generation sequencing data have a high proportion of false detection due to errors in base calling, alignment, or *de novo* assembly, especially in repetitive sequences that cannot be spanned by short sequencing reads. Long read lengths obtained by single-molecule sequencing techniques have recently been employed to discover structural variants in human samples to overcome the disadvantages (Chaisson et al., 2015; Pendleton et al., 2015). However, the high cost and low throughput of this method now prevent it from being widely used.

In contrast to phiRv2 (RD11), phiRv1 (RD3) is nevertheless of interest despite the fact that it cannot be considered a stable RD. It is thought to be incapable of encoding infectious phage particles (Fan et al., 2016), but may still have an active integration/excision system that enables transposition in the genome, and we found suspicious transposition-like phenomena in some strains, similar to IS6110 with multiple copies in the genome. We discovered breakpoints at the original position of RD3 using structural variation detection, indicating its deletion, although sequencing coverage revealed that it was not absent. Correspondingly, we found breakpoints in these strains indicated the translocation of RD3 (insertion sites 103.7 and 388.4 kb) which was comparable with previously observed RD3 insertion sites (Bibb and Hatfull, 2002).

A mobile element such as IS6110 is one of the causes of the genome assembly gaps. The IS6110 insertion found in TbD1 in the genome of lineage 8, prevented us from detecting the whole TbD1 in the assembled draft genome. And recombination between adjacent IS6110s might result in deletions of regions within, and such deletions were difficult to be detected due to the difficulty of identifying the accurate breakpoints. The presence of mobile elements or repeat sequences at either end of deletions represented the unstable region in MTBC genome, and deletions in these regions might occur independently in strains of different lineages and had inconsistent deletion ranges. This may explain some of the overlapping ranges of RDs that may result in some RDs being reported as missing in strains from non-specific lineages.

Although we have found RDs or RvDs accordingly in most of the evolutionary branches, it is still difficult to determine whether these deletions played a key driving role in the generation of these lineages at this stage. Deletion of the TbD1 increased the resistance of MTB to oxidative stress and hypoxia and enhanced its survival in granulomas, which is one of the primary drivers of modern lineages globally widespread (lineage 2∼4) (Bottai et al., 2020). We assume that the absence of some RDs and RvDs may have given MTB strains a competitive advantage in transmission, and pathogenicity, leading to the formation of new lineages. Alternatively, the partial range overlap of RDs that occurs independently in strains of distinct lineages might be the consequence of convergent evolution due to the same selection pressure. It’s unclear why distinct deletions of the RvD4496 occurred in various evolutionary branches, but its intricate deletion in lineage 5 demonstrates limits beyond those of a single reference genome. The structural variant identification software Giraffe, developed by Sirén et al. (2021) provided good insights into the ability to map sequencing data to multiple reference genomes simultaneously to obtain more diverse and accurate genotyping results. The simultaneous use of reference genomes from multiple different lineages, including H37Rv, may help identify more structural variants associated with MTBC lineage differentiation.

Moreover, in comparative genomics investigations with NTM, *M. canetti* had earlier found the deletion of the *cobF* region in the MTB genome (2013) (Supply et al., 2013), which might result in MTBC’s inability to synthesize vitamin B12 like other mycobacteria, which could be taken as a foreshadowing of lineage 8 discovery (2020). It can be assumed that in lineages with multiple RDs, the deletion events do not occur simultaneously and therefore intermediates may still be present. In the evolutionary branch with deletion RD7-RD8-R10, hosts jump between humans and animals may have occurred during evolution repeatedly, and the discovery of intermediates may assist in understanding the evolutionary history and the interaction of MTB with the host (Brites and Gagneux, 2015).

## Conclusion

We implemented multiple methods to search structural variations in the MTBC genomes and identified 125 RDs and 5 RvDs, including 34 newly identified RDs (RD501∼RD527 and RD2_ER while RD502 and RD516 contain multiple deletions) and 2 RvDs (RvD533 and RvD4496), specific to distinct phylogenetic groups. Thereinto, unreported RDs and RvDs were discovered in several new lineages and recognized sub-lineages. Further, we examined the distribution of RDs and RvDs in MTBC, and the results suggested that most of RDs and RvDs are persistent traits in parts of the MTBC lineage. Analysis of the RvD4496 region for lineage 5 revealed a complicated deletion, demonstrating the limitations of using only a single reference genome in comparative genomics research. The distribution of partial RDs with overlapping ranges in the phylogenetic tree implied that convergent evolution might result in the absence of identical regions in the genome due to exposure to the same selection pressure. Furthermore, as this study was performed *in silico* and the results need to be validated experimentally and evaluated using a dataset with additional samples.

## Data availability statement

The datasets presented in this study can be found in online repositories. The names of the repository/repositories and accession number(s) can be found in the article/Supplementary material.

## Author contributions

QS and ZL designed the research work. ZL and ZJ performed the research activities and wrote the manuscript. ZL, ZJ, and WW analyzed the data and validated. QS, WW, XX, YM, and XG edited the manuscript submitted. All authors have given their approval for publication.

## Footnotes

1
https://www.ncbi.nlm.nih.gov/sra/

2
http://www.ncbi.nlm.nih.gov/books/NBK158900/

3
https://github.com/shiquan/bamdst/

4
https://github.com/farhat-lab/fast-lineage-caller/

5
https://tandem.bu.edu/trf/trf409.linux64.download.html

6
https://itol.embl.de/

7
https://github.com/tseemann/shovill/

8
https://github.com/soedinglab/MMseqs2
