# Supplementary material for: Identification of region of difference and H37Rv-related deletion in Mycobacterium tuberculosis complex by structural variant detection and genome assembly
Source: Front Microbiol. 2022 Sep 8;13:984582. doi: 10.3389/fmicb.2022.984582 (PMC9493256; doi:10.3389/fmicb.2022.984582)
Supplement: Supplementary file 4 [file Table_4.DOCX]

**Supplementary Material 4**

Figure S1. Deletions in the RD14 region of multiple lineages.

Figure S2. Deletions in the RD5 region of multiple lineages.

Table S1. Gene covariation in RvD4496 and adjacent region of lineage 5, lineage 8,

*M. canetti* and H37Rv.


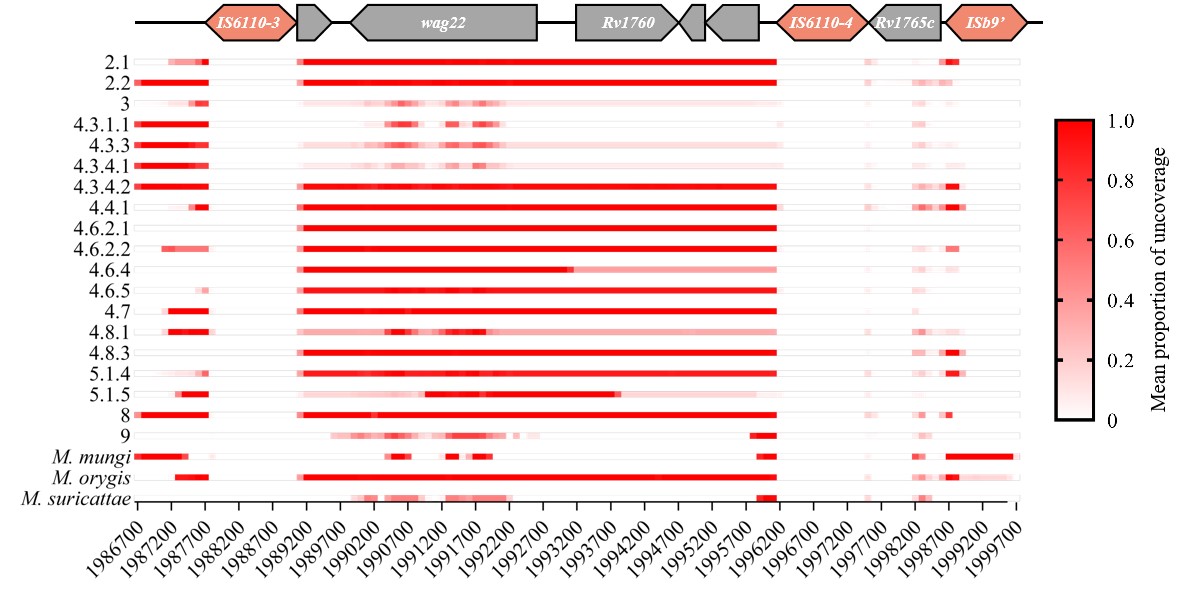


Figure S1. Deletions in the RD14 region of multiple lineages.


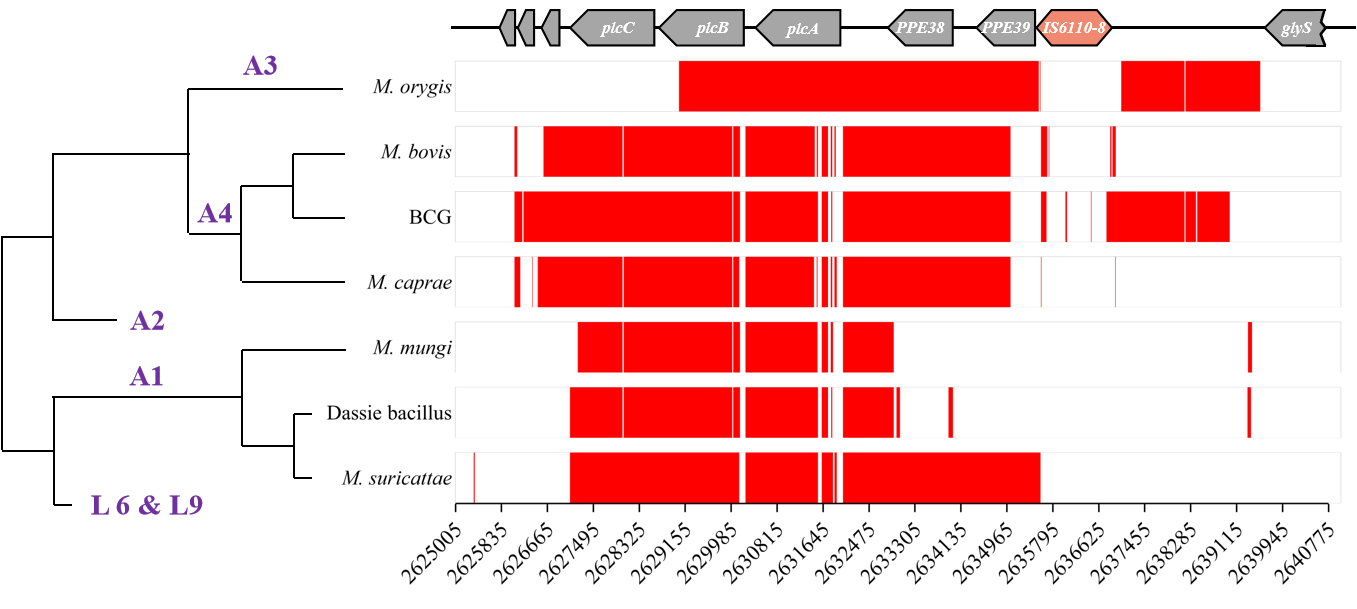


Figure S2. Deletions in the RD5 region of multiple lineages.

Table S1 Gene covariation in RvD4496 and adjacent region of lineage 5, lineage 8, *M. canetti* and H37Rv.

| Lineage 5 | Lineage 8 | *M. canetti* | H37Rv | Orientation |
| --- | --- | --- | --- | --- |
| *JQ847_RS10325* | *tig002_RS10310* | *MCAN_RS10390* | *Rv1976c* | - |
| *JQ847_RS10330* | *tig002_RS10315* | *MCAN_RS10395* | Deleted in H37Rv  (RvD4496) | - |
| *JQ847_RS10335* | *tig002_RS10320* | *MCAN_RS10400* |  | - |
| *JQ847_RS10340* | *tig002_RS10325* | *MCAN_RS10405* |  | + |
| lineage 5 deleted region | *tig002_RS10330* | *MCAN_RS10410* |  | + |
|  | *tig002_RS10335* | *MCAN_RS10415* |  | - |
|  | *tig002_RS10340* | *MCAN_RS10420* |  | - |
|  | *tig002_RS10345* | *MCAN_RS10425* | *Rv1977* | + |
|  | *tig002_RS10350* | *MCAN_RS10430* | *Rv1978* | + |
|  | *tig002_RS10355* | *MCAN_RS10435* | *Rv1979c* | - |
| *JQ847_RS10345* | *tig002_RS10360* | *MCAN_RS10445* | *mpt64* | - |
