# Supplementary material for: Identification of region of difference and H37Rv-related deletion in Mycobacterium tuberculosis complex by structural variant detection and genome assembly
Source: Front Microbiol. 2022 Sep 8;13:984582. doi: 10.3389/fmicb.2022.984582 (PMC9493256; doi:10.3389/fmicb.2022.984582)
Supplement: Supplementary file 5 [file Table_5.DOCX]

**Supplementary Material 5**

Congruent sequences of RvD2 and RvD5 in different genomes.

>RvD2

GTTCTGCGGCATGATGCGCCAACCGAATCTCCGCACCGGTTGGAAAGTGGGACTCTGCAGCTCCGGCCCACCATTTTGGCCGTCGGGGTCGATGGTGGCGCTCAGCCAATAAAGGCGGTTGGGCAGGGTGGGACCCAATACCGAGCAAAAGTAGCGGTCGCAGACGGTGAACGCGTCGGCCAACAGATAGTGGATCGGAATGTCTTGGCGCGTGTAGTAGCCCATCACCGTGGGAGTGTGGGCCGCCGAGCGAGTCTTGGCCTGCGCTGGTAGCCAGTTGTCGTTGACGCCACCATTCCACGACTCATGCATCGCCACCCAGCTATGGTCAGGGTCGTTGACACACGCGCCGTCGAGGAACGGGCCTCGGGTGGTGTCGAAGCGGTAGGGCATCGTAACGCCGGTGGCGTCAAGAGCCTGCGTCATCGGGTTCCAACCCTTTTGTTGGAAGAGCGGCGATACCGTGTTGAATCCATCGGTGCCGGAGAGTGTTCCGAAGTAGTGATCGAATGAGCGGTTCTCCTGCATGAAGAACACAAAGTGTTCGATGTCGGTCAAATGGCCGGAGCAGGGCCCCGCGCCGTAGGCCTTTTCAATCACCGGACCGGCGAAAGACATCAACGCGCCGGCGCCGCCCGCAGCGACCTTAGCTAGGAATTCTCGCCGCGAAACTCCGCCGATGTGGCTTTGGCTCACCGCTGTGTTCTCCTGTCGAACCTCCAGCCGCATTTCAGCTCAAGGTAGAGGACTCCGACGAACGATCACGACGCGCCAACCGGCGTGTCGCGCGCGAACTTGCGGATTTCGGCCGCAAACTTGAACCGCTATGCGGTGTCTTGGGTGTCGCCGCACCGGGTGCTTACGGCCATGGCGCCCAATGTTGCTAGCATGCTGGCTGCTGCCCGTTGCCAGGTGAATATTTCGGCGCGGCGGCGCGCGCAGCGGCGCCGGTGGCGTTCGGGCCGGCTGACGATTGTGCGGACTGCGTGTGCGATGGCCTCTGGGCGGTTGTCGGCGCAGGCGCCGCTGTCTGCGGTGATGATCTCGGTCAGCGCCGAGGTGCGGGACACCACGGCCGGTGTGCCACACGCCAGCGATTCGAGTGCGGCTAGCCCAAATGTCTCGTGTGGCCCAGGTGCCAATGCGACATCGGCCGATGCCAGCAGGCCAGCGACGGCATGCCGATCCGAGATGAAACCGGTGAAGTCGATCGGCAACCCGGTTGCCTTGCGTTCCAGCCTGGCGCGCAGCGGACCCTCGCCAGCGATGACCAGTCGAGCGTCGACGCCGGCGTCACACAATGCGGCGAGTGCGTCGATGCTGCGGTCAGCGTGCTTTTCCACCGACAGCCGGCCGCAGTGGACCAGCAGGATCTGCGTCGGGGTGGCCCAGTGCTGCCGAACCCGGGCACAGCGCCGCCGCGGGTGGAAGGTCTTCAGGTCTACGCCCAGTGGGACGGTGACGGTATTTGTCGCTCCGATGCGGTCGAATTCTTCGCGCGCGAACCCGGTAGTACACACGACAGTGTCGTAGTTGGCGGCGGTTCGCGCGTTGGCGAAGTCTGCGAACTTCTGCGCGGCTCGACGCGGAAGCAATTGGCCCGCAAAGCGATCAAGACGCTCGTGGGAGATCATCACCGTCGTAACGCCGTGTTCGCGGCCCCACCGGCCCAGTGACCTCAGGGTGAGCCGGTCGGAGACCTCCAGGGTGTCTGGTCGCAGTGTTTCCAATACAGTCCGCACGGCTCCTGGCATAACCGCGCGATAACCACCGGTATATGGAATATGCTTGGCGGGCAAGGTAATTCGAACAACACCCGTGCGTAGGAGGTGTCGTTCGGTGCGCGCCCCCGGGACGATCAAAACACCTCGTGTCCGCTGGCGCAGTATTCCGCGCCCAGCCGGTCCACCGCGGTGCGGAGTCCGCCCGAGCGAGGTCCATAGAAGTTGGCGACCTGAACAACACGCATACCGTGAGCAGAACCGGCCGTCGTGTGCGGTCAACGACATAGCATCGACGGTTCCCTGAACGGACCATGAACTCCTGCGGAGCGGGCACCTGCCTGCGCTTCGCGCCAGCCGACAGACACAACCAGAACTTGTGAGCGCACAAGGTCAAACCCGCTACTGGAAGTTCGAGCAACGGCGGAGCATGGGAGTTGACCGATCGAGGGGAGAACAGGACAACGATTGCCATGCTGGAGACCGCTGGATTATGGGGCAAGCGCGCCGACATGATTGTGCGTGGATGCTTGCCTTATAACGCTGAGCCACCGCCGGCCGTGTTGGCTGGCAGCGACATCACCCCGATCAATGCGTTCTACGTCCGCAATCACGGCCCGGTCCCCGACATCGCGCCGCAGCATTGGCGGCTGACGGTCGGCGGGCTGGTGGACAACCCGCTTACCGTGACCTATGAACGGCTGACCACCGAGTTCGACCAACACTGTGTGGTGGCGACGCTGGCGTGCGCCGGCAATCGGCGTGCGGAGCTGTTACGGGTGCGACAGATCCCAGGTAAGGAACCCTGGGCGCACGGTGCGATCTCGACCGCTCAGTGGTGCGGTGTCCGTCTGGCAGACATCCTGCAGGCCGCCGATGTGCATATCGACGAGGGGCTACACGTGGCCTTCGATGCTCCGGATGTAGCTGAGGAGGCTCGCCCCATCCAGCCCTATGGCAGCTCGATCCCGCTGAGCAAAGCGCTGTCGCCGGAAGTTCTGCTGGCCTGGCAGATGAACTCCGAACCGCTGCCGCGTGCCCACGGTGGTCCGGTTCGCGTGGTGGTACCCGGATTCATCGGGGCCCGCAGCGTCAAGTGGGTCACCGCCATCACCGTGCAGCCTGGTGCTTCGCAGAATTACTTTCAGGCTCTGGATTACCGCATCCTTCCGGCGGATGCGGACGCCGACATCGTCGGGCCGGGCGAAGGGATTTCGCTTTCGTCGCTGGCGCTCAACTGCGACATCCTCGACCCCACCGATGGCGACGACGTACCGGCAGGGGCGCTGACCATTCGTGGCTATGGGATGGCCGGCGATGGCCGCAGTGTCGAACGAGTTGATGTCTCTGTCGACGACGGGCTCACCTGGCAGCAGGCCGACCTACACGCCGCGCCCAGCCAGTGGTCATGGCGGCCGTGGTCGCTGACGGTCGACGTGGAGCCGGGGCCGTTGGGTATCACCGCACGTGCCTGGGACGATACCGGGGCGCTGCAGCCCGAATCGGCTGTGTCCCTGTGGAATCCGCGCGGATACGGCAACAACGCTTGGGCCCGCGTCGCATTGCGCGTGAGTTAGCCGGGTACTCGGTCATCAACCGGTTGCGGGGCCCTCCGAAGACCACTGGAAAGCACTGCCGATCTGATGGTTAGGGTGGTTGAATTAGCCGACTCGGTCGGCGGTGACGCCCCGAGGTCAGGTGAGGCGAAGGTGATGCCAGTTGACGGAACTAGCCGGCGACACGATACCCGACCGGTGGCTCTGCTGAGGCCGACGCGGTGACCGCCATTGGACGACTTATCCATCGCTACGCGATATGGATCGTCGGCGTCTGGGCGCTCGCGGCCATCATCGGGAATAACTTTGCCCCGCCACTCGAGCAGGTCATCACCGCCGAGGATCAGCCGTTCTCGCCGGCTGGCACCGCCACTTCGCGTGCCGTGGAACGGTCAGCGGCGGCCTTCTCCCAAGCGCCCGGCGACAACATCGGATATCTCGTGCTGGAGCGAAACGGAGTCCTCAACGACCAGGACCGGGCTTACTACGATGCGCTGGTCGTGGCCCTACGCCGTGATTCCCGCCACGTCATCGAGGTGGTGGACTGGTGGGGAACCCCGGCCATCGCGGAGGTCGCCCGCAGCGACGACCATCACGCGGTGACAGCTGCCCTGCGCTTTGGGGGCATGGTCGGAACGTCGCAAGCCGGGGAGTCGATAACCGCCGCGCGCAGCATCGTTACCCAACTGCATCCCCCCGACGGTTTGCACGTATTCGTCACCGGTCCTGGCGCCACCATCGTGGACGAGTTCGCGGCAATCGACAGACAGACCCAGCTCATTACGGCAACGACAATCGTGGTGTTACTGATCCTCTTGTTGATCGTCTACCGATCCGCGATCACCGCGACGGTGCCGTTGTTGTCGGTCGTCGTTTCCCTAGCCGTGGCCAAGCCGATCGTTTCCGTCCTTGTCGACCGCGATTTCATCGGGATATCCCTGTTTTCCCTCGGACTTAGCGTTGCGGTGGTTGTCGGCGCGGGAACCGGCTTCGCGATGTTCCTGATCGGGCGTTACCACGAACGACGAAGGCAACATATTGCCCCGGCGGCGGCGCTGGCAGACGCGTACCGCGGGGTGGCGCCGGCGATCGCGGGTGCGACGTTCATCGTGGTCACATCGCTGGGCGCTGTGGGATGGCTGAGCCTGGCACGGATCGGTATGTTCGCAACAACCGGAATCCTTTGCTCGATTGGCGTTCTCGCAGTGGGCCTGGCCGCACTGACGTTGACGCCAGCTCTCGTCGCGCTGGCCAGCCGTGCCAACCTCCTCAAACCGCCACAACACAAGCGCATACAGCGCCAATTTCGGCGACTCGGCACACATGTGGCGCGCTGGCCGGCGCCGATATTGGTAGCCAGCGGTGTGTTCGTACTCATCATGATGATCGCGCTCCCTAGGGTGCCGATCGGCTGGGACGAAGCCGCGGCAACCCCGTCCGCGGCGGAATCCAATCGCGGTTACCGGGCGGCCGATCGCCACTTTGCCCCGAACCAACTGCTGCCCACCCAGGTGATGATCGAGACCGACCACGACATCCGCAATCCCGCCGGTCTGACCGCGATCGAACGAATCACTGCCGCGATCATGGCTATTGGCGGTGTGCGCATGGTGCAGTCGGCGAGTCATCCCAACGGAATGGTGTCCAAGCAGGCTGCCTTGACAGCATCGGCGGGGAATCTCGGTGATCAGCTCGACGAATTTTCCGATCAGCTCACATCCAGGCAGGCAACGTTCACCAATCTCGAAGCTGCGGTCCGCGACGTGGTGTCAGCCCTCGATCTGGTTCAGGCTGGCATACGACAGGATGGCTATGGACTTGGCCAGGTCAGTCTGGCCGTCCGGCTGATGCAACAGGCGATAACCAAACTTCAGGGCAGCGCCGGTGACGTCTTCGACATATTCGACCCGTTGCGTCGTTTCGTCGCGGCGATACCCGAGTGCCGGGCCAACCCCGTGTGTTCGGTCGCCCAAGAGGTGGTGCAGTGGGCAAACACCGTCACCGAGAGCTGTGCGAAGCTGGCCGATGCGGCAGGGCAGCTCGCGCGGGGGATCGCTGATGTCGCCTCGGCGACATCGGGTGTGTCCGGGCTACCGAATGCCCTGGACGGCATTGGAGGTCAGCTGGCGCAGGTACGAGAATCGGCCGCAGGCGTTCAAGAGTTACTTAACAATGTCGGCGCAGCACCATTGCGAGAGCTTCCCGACTATTTACGCGAACTTGCCGCCGTCTCCCAGAGTGCGCCGGGCGTGGATCTCTACGCCGCTCGGCGAATTCTGACCGACCCGAATATGCGCGCGGTCTTGGACTATTTTGTCTCACCAAACGGCCATGCAACGCGTTTACTCGTCTACGGCGACGGGAGCGAGTGGGGTGACGATGGCGCCCAACGCGCTCGCGCGATCGTGACTGCGGTGGCCGAGGAAACCGACGAGGGCACGCTGCGACCCACCGCTGTTGAGCTGACCGGCGTTGGACCGGCTACCCGTGACCTGCAGGATCTGGTGGGCAGTGACCTGACCTTGCTGGCGGTCATCACACTGGCCGTTATCTTCGCGATAGCCGCACTGCTGCTGCGCAGTCCGCTTGCCGGGCTTGTGGTCGTCGGCACAATCGCGACATCGTATATCTGTGCGCTTGGCGCCAGCGTAGTGATTTGGAAACACATACTTGGCGATAACTTGCACTGGTCGGTATTGCCGATTGCGTTTGTTTTGCTGATATCGGTGGGTTCGGCCTACAACCTGCTCTTCGCGCTGCGCATCCGCGAAGAAAGTCCTGCCGGGCCACGAACCAGTGTCATCCGAGCGTTCGCGGCGACCGGAATGGTAGTCACGGCCGCTGGAATCGTGTTTGGCACAACGATGTTCGCGCTGGCCGCGAGTACCTCGCTGAGCGTGGCACAGATCGGCGTTACCGTTGGCATGGGGTTATTGCTGGACGCCCTTGTGATACGAGGCTTTGTCCTGCCGGCCCTGATGGTTTTGCTGGGCCGCTGGCTGTGGTGGCCGCGCCGATCGGTTAGCAACCGGCAGGTACCCGAGCCGTCGCCGGCCTAAATTGAACCGATTCACGCGTGCATACGTATCCGAGAGTGTGACGAGCCGAAGCGCAGCAGCCGGCTAGGAGCTTCGCTGTCGGCCGAAGGCTGGGTTGATGCCTGGGCCACCGCAACCCGCTGCATTTGCGCACCGGACAGCACCTTAGCTGTGCACCAGGGCGACGACCCGTGCGCGACCGGTCGCCGAGATGGGATACCCGAGCCCGATCCCTGGCCGGTTCGGCCAGGCACGCCGGACCCTCCTGGTGTGGGAATGTGTCCAAAGGGCATTATTGGTCCGTGAGCGCATTCAACATCTGTCGATTCGTTGGTGCCCCAATGGTCACGACGTGGGCACTGCTGTTCGCGCCCGTACCCGCCGCGTCTGCCGACCCACCCGACCCGACGGTATCGGATGGCGCGTGTCCCGATGTTGAGGTGGTTTTCGCCCGCGGGACCGGCGAGCCACCCGGCGTGGG

>RvD5

AGATATCGGGGTGATGATTGAATTTGTCGGCGATGGCGGCGATCTTCGCCACAAATTTCATGGATTGATCGAAGCTCCCAAACCCAAATGTGTGGCGGAGCTTTCCATCGACGAGCTCCCACCCGGGCAGTGCCGTCAACCTCTCGGACAGCTCCCTGTCGGTGAGAGCCGACAGCCTGCCGCAGCACACGTCGTGAAGGCTTGACTCCTCACATCGACTGACGTTGCCGTGTGCCATGGTTCCCTCCTGGACTTGGTTGGATTGATGGTGGATGTCGCACCTGGTCGCCCAGCGAGTGCGCGCATGCGGGCATGGGCACCAGACCGGCCGGAATTATCCGCCTCTGGTGTGCATCTCCAGATGTGGGTCGCGTTTCAAGCGGGACACCGGAAGAAAGACCTGTCGCGTTCGTTGGGCCAAGCGTTGTTCTGCCCCACGGTTCGCGCGGTCCCGCCAGCCACGCTGCAGAATCTGCACAACGTCATTGGCGGACGCTCCCGCACGCACGGATTCCCGCAAATTGATGCCGGACAGGGCGTAAAGGCAATGTAGCCAGATGCCATCGGCGGTCAGTCGCGACCTGTCACAGGTTGCACAGAATGGCTCGGTGGTCGAAGCAATGATGCCGAATGTTGTTCCGTCCGGCAATCTGTAGCGGTTCGCGGGCGCGGAATCGTACTTCGGCAAGGCCGCGATGGGACCGTATTTCTTCCCGAGAGTTGACAGCATCTGCGCTTTCGTGAAGACCTTGTCCATCGACCATTGCGTCGCACCGCCGACGTCCATGTATTCAATGAATCTCACCTCGGCATTGACGTTGCGAGCGAATTCGATCAGATCGGACAACTCATCATCGTTGAAGCCACGTATCACCACGGAATCAAGCTTCGTATCGGTGAAGCCCGCCGCCGCAACCGCCTCAATGCCTTCGATAACTTTATAGTGGGTCCCGCGCTGGCTGATTGCTTTGAACCGGTCCGGCCGCAACGTATCAAGACTAATGGTGATGCGCCGCATGCCCGCCGATTTTAACTTTCTGGCCTGGTCCGCGAGCAGCACGCCATTAGTTGTGATCGCCAAATCCTGTAAACCGCTGCCATCGCCAACTTTCGCGCTTATAACCTCAATGATTGCCGCCAGATCCGAGCGGATCAGCGGTTCTCCACCGGTGAGCCGAATTTTATCGACACCGACAGCGATAAATGCATCGACGATCAGGCTGATTTCGTCCACAGACAGCAGATCCGCCCGCGGCAACCAGGCGTACTCGGCCTCGGGCATGCAGTAGCGGCAGCGGAGGTTGCACTGATCGATCACAGAAAGCCGCAGATCACCCATGGTGCGACCACAGCGGTCCCTGATGGGGGATTCATTGATGCACAAGCCGGACCGGCTCACCGAGGCGACATCCGTGATCTGATAGCGCGACTGTTCCGGTCCGGACAATGCTGGATGAAAAACCCGAGTCATTGGATAGCTCCGCCGACAAGGCCACCGACGGCGATGGGCGACGCCAGGCATCCCCGCAGGGATCTAGGACATCGGGCGTTATCGGGGGCTGGCCGCGGCGATGGCGCCCGCGAAGACCATCGCAACACGTTTCGCGTGCACACTGGCACCACATGCCTCCTTCTCATTCGCGCGCTTTCCGGGGTTCACGTTCATTACTGTTCAACACCGGCCTCAATGGAATCTCAATGCGCACGGCGCACGACCGGATTCGGCGGCGCATGTGGGTCCGGCAAGGCAACGGCCACCCGTTGGCCGTTCATCCACGACGGTGACCCGAATTCCCGACAGTGATGTCGAGAAGCCAGGTAGCTCCGGTTCGGTGTCGAGCACCGGGCCGGAGGTCGCCGCACGGCGTCATGGTAGGCGTTGCGGGTGCGACCGCAATCGGTCACCGCCACGTTCCAAATCGGTCACACTTGACACCCGTCATGTCGGCGGAGCCGGTATTCTTCGAATTCGAGCTTTCCCCATCAGGCCCTGGCGCCCGGTGAATCGCTCATCAAACGGACCCGCGACCGACGAGGATTGTGATGGCCCGAAACGAACTCCGGTTCGGTATTCTCGGACCGCTAGAGATAAGCGCAGGTTTCCGCAGTCTACCGTTGGGCACACCGAAGCAACGTGCGGTCTTGGCGACGCTGATCATTCATCGTAATCGCCCGGTTGGGATCGACTCGTTGATCGACGCGGCTTGGGAGCAGGACCGGCCGGAGGGATCACGAGCGACCGTGTATACGTATGTCTCGAATTTGCGTCGGTTGGTAAGCACCACGGGGGCGGATTCGCACAGCATCTTGGCTAGCGCACCGCCAGGGTATCGACTCGCCGTTGCCGATAACCAATATGATGTGGCACGTTTTATCAGCCAAAGGTCGGCCGGGCTGCGCGCCGCCGCTGCCGGTTCTTTCGAACAGGCCAGTGACCATTTGTCGGCCGCGCTGGCCGAGTGGCGCGGCCCGGTCCTGGACGATCTGCGTGAATTCAGCTTTGTCACCCGCTTGGCCAACTCATTGGTTGAAGACAAAATCATCGCCCACACAGCTCTCGCGGAGGCTGAAATCGCCTGCGGGCGTGCCGATTCAGTGATCAGCGAGCTCGAGGAGCTGATCCTGGAGCATCCTTATCACGAGGCCCTGTGGCGGCAACTCATCGCCGCATACTATGTCTCGGAACGTCAATCCGATGCCCTGGACGCCTACCGGCGATTAAAGACCAGCCTGGCCGAAGACCTCGGCGTCGACCCGGGACCCAAGGTACGCACGCTATACGAGCAAGTGCTGCGCCAACAAGCACTGGACACGCGAGTCGTCGTCCAGGCTGCCGCAGGAGATATCATCAGGGCCCTCGAACACTCTCCCGGCATGACCGACCGTTCGCCGCGCGCCGCAATACGCGACGCCGCGGGGCACCGGTCTCCACTTGGCCGGTTGCCCCTTCGTATCGGGCGTAGCAAAAGTAACGACATGGTGCTGCCGGACGGCAAAGTCAGTCCCTACCATGCCGTTATCGTCAACACCGGTGAAAGCTTCATGATCACCGACCTGCGATCGGTCAACGGCGTCTACGTGCGTGGGCGGCGCATCGCGACCACAGCCACCCTCAACGACGGCGACCACATTCGCATCGGCGACCATGAACTCACGTTCGAGGTCATACCGCACGAATCGGGCCGTTAGCCGGCGGGTTTGCCCATCCCCGGTTCTGCGCCCACCCGCGCCTACCCTTCGGTAGGCAGGCCGGCTCGCCGACCCGCCGCGGACACCAGGATGCCTACCGTGCTGGCACCAGCACACTCTCGTCGTATTGCCTCACCAGGTCTTCGACCACGGTCTGCCACGAGGCATCGTCCACGCAGAGGCGGCTGGCGGTAAGTACTAGCCGGTCGGGACTTCCGGGGAGCAGAGAAACCGCGAACAACCTGCCGGTGTACATATCGAAGCTCGCGCTGTGCCGTGCGATCACCTCCGCGACGGCGGCTCCCGGGGGTTCCACTCCCCAGCCCCACCCGCCGCCGCCCGGGCGAGAAGTCAAGGTGTCAACGCGCGGCTCGAATACGGTGCCAAGGGCCGGATGGGCGTCGAAGACCGCCTCCACCGCCGCGCGGATACGCCCCTGGTCCACCCGTGAGTTCGCAATCAGAACGTCGGTGTGCGCGCCAGGCTCGATGTCCTCGAGCCTGGGAAGCTCAACGAAATACGAATACGACATGACCACAGACTCCGGCACACCCCTCAGCGAATTCTTGGCAACTTCTTGGACCTGGGCCGAGCAGACGCAAAAGCACCCCATTTCGGCACGAAATGGGGGCCCTTTGCGTCT
