# Supplementary figures and images for: Identification of region of difference and H37Rv-related deletion in Mycobacterium tuberculosis complex by structural variant detection and genome assembly
Source: Front Microbiol. 2022 Sep 8;13:984582. doi: 10.3389/fmicb.2022.984582 (PMC9493256; doi:10.3389/fmicb.2022.984582)

# Supplementary Material 6

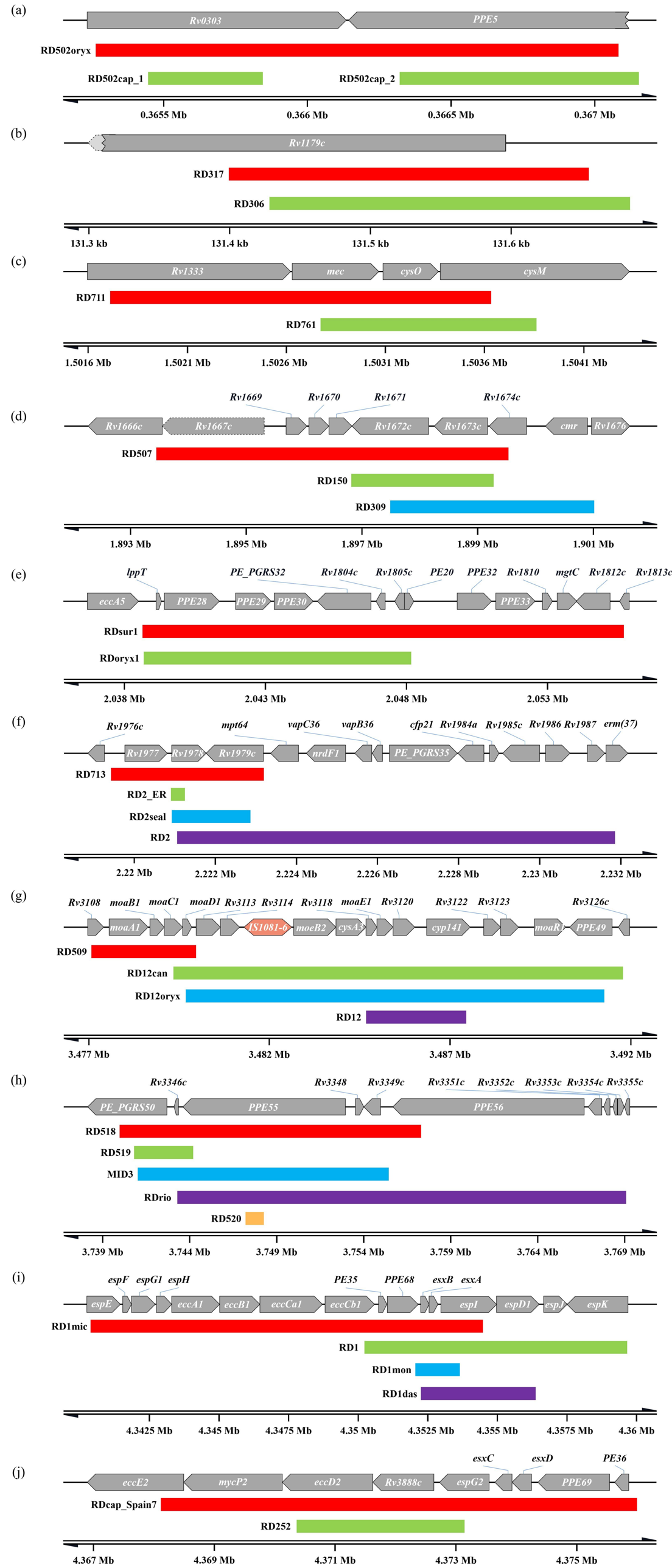

Supplement: Supplementary file 7 [file Image_1.PDF]

# Supplementary Material 7

(a)

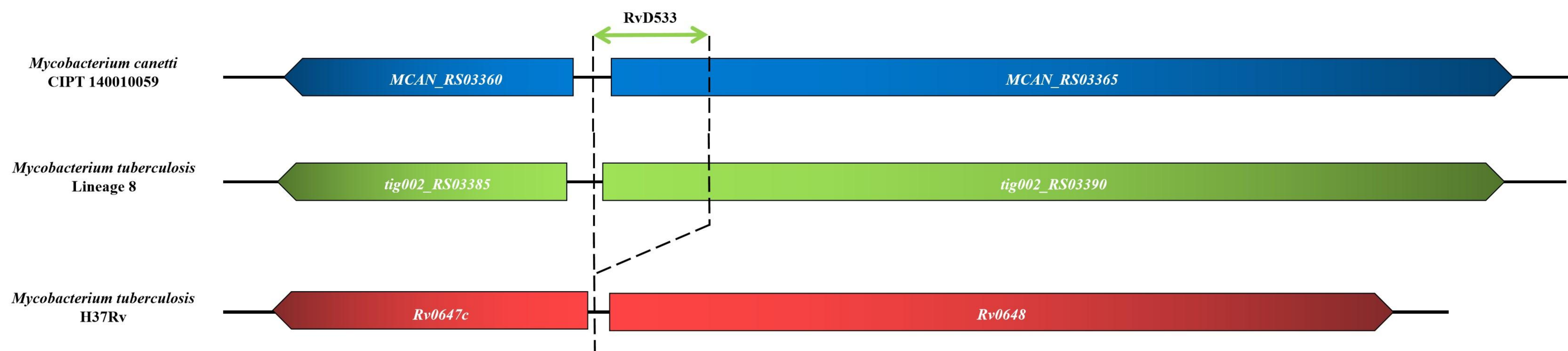

(b)

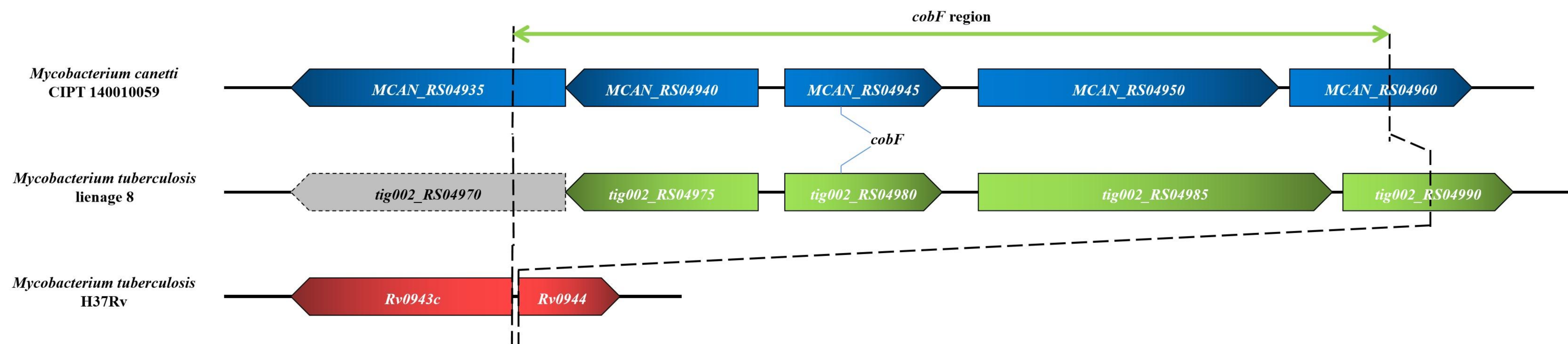

(c)

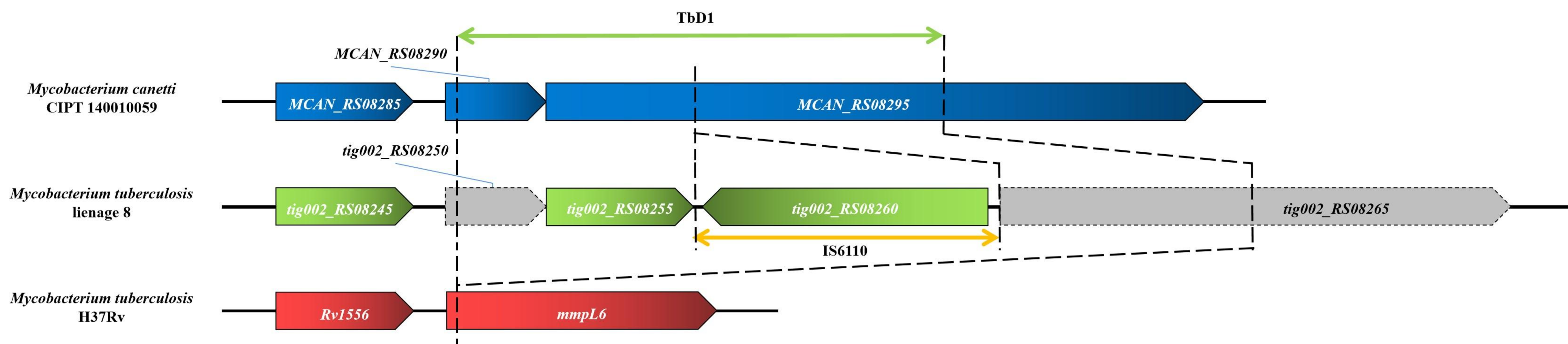

(d)

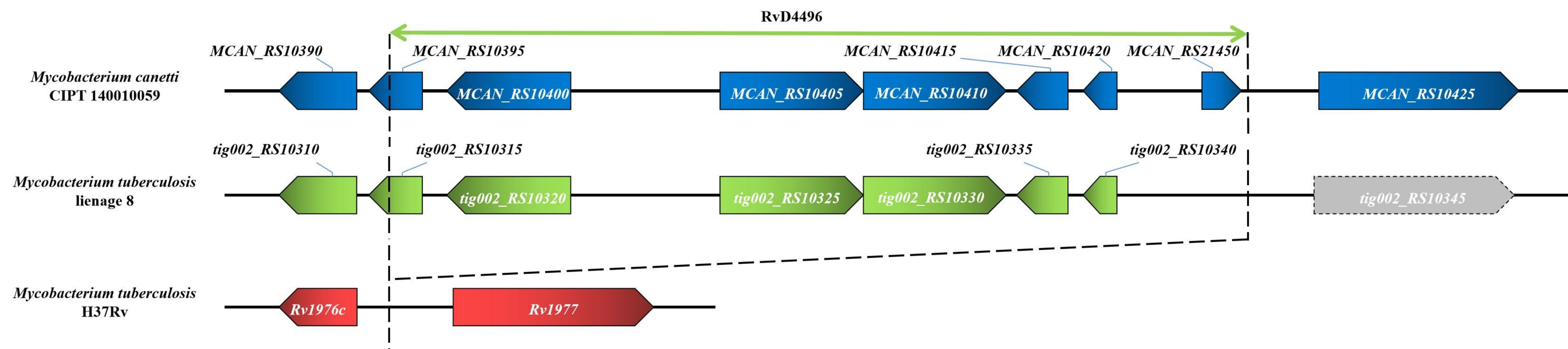

(e)

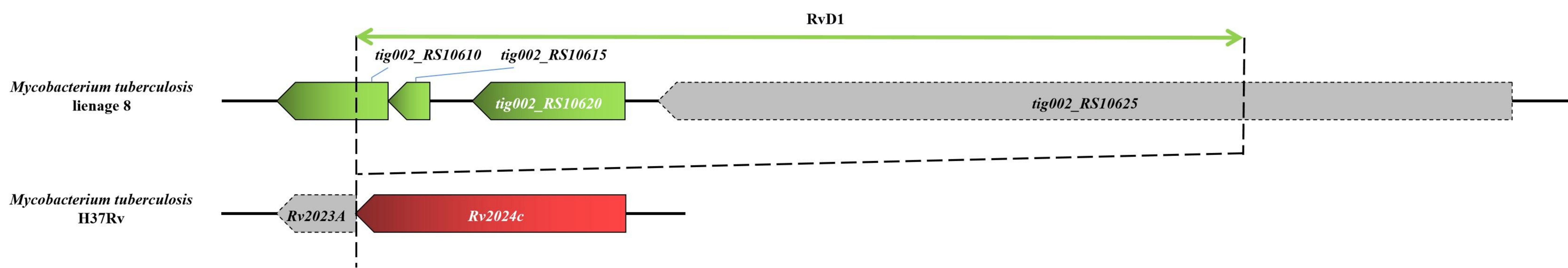

Supplement: Supplementary file 8 [file Image_2.PDF]

Supplementary Mterial 9. The work flow

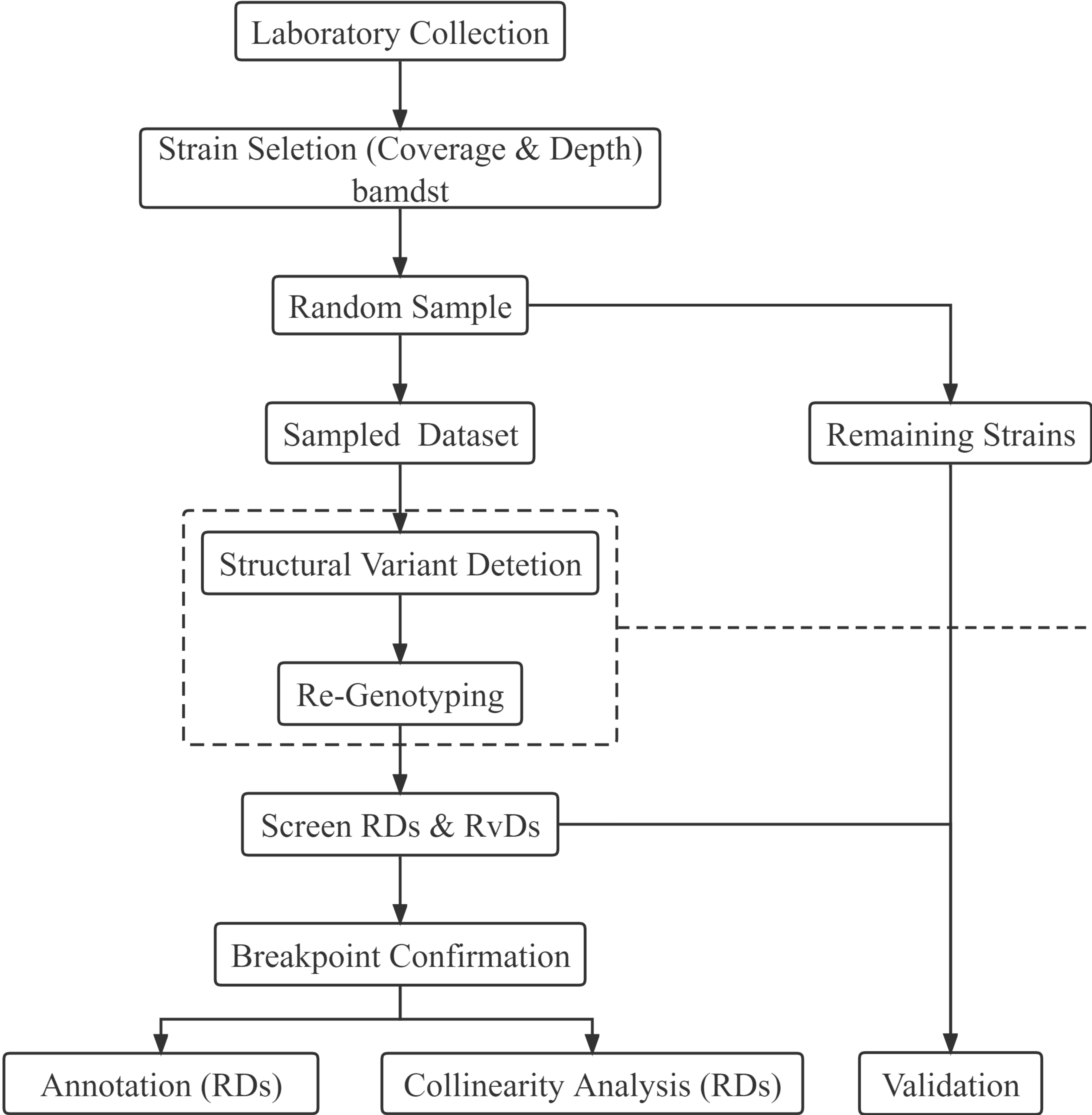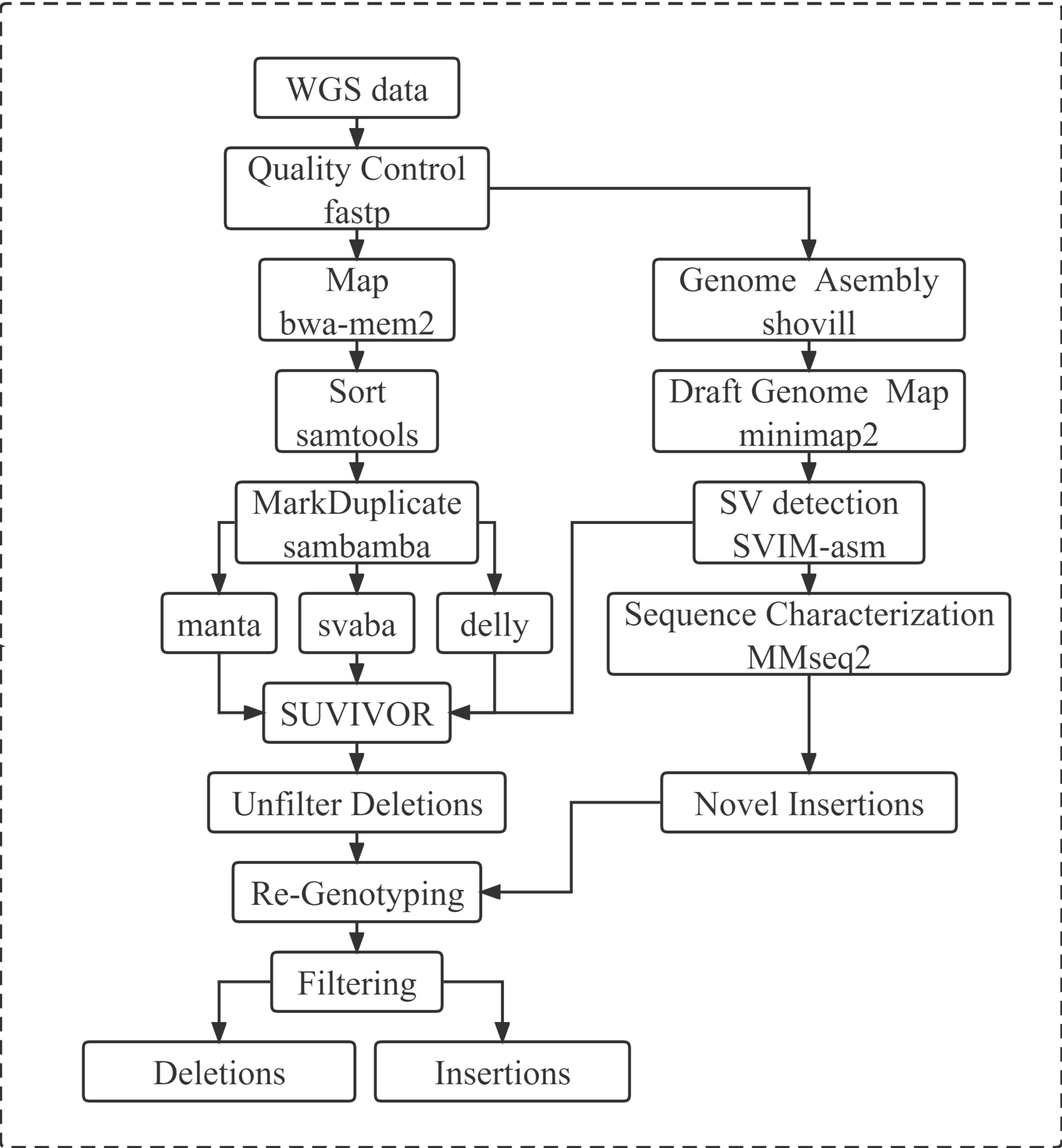

Supplement: Supplementary file 9 [file Image_3.PDF]
